# Some Cases of Fever

**Published:** 1826-12

**Authors:** C. Hewett

**Affiliations:** Physician to St. George's Hospital.


### FEVER.

*Some Cases of Fever, treated upon the Principles lately explained by* Dr. C. Hewett, Physician to St. George's Hospital.

Case I. *Fever, with Pulmonary Inflammation.*—Brian Flaherty, ætatis twenty-seven, was admitted into St. George's Hospital, July 24th, 1826. He has been ailing for the last six weeks, with slight cough and rheumatic pains; for the last three days he has had giddiness, headache, and other symptoms of fever. This morning he has been shaking with cold, but the skin is now pungently hot and dry. Any attempt to take a full inspiration excites cough, and pain in the left side of the chest: the latter is said to be felt only now and then, and does not prevent him from lying upon the left side; the cough, which was previously moist, has latterly become quite dry. The eyes are watery and suffused; pulse is 116, and not corresponding in strength with the temporal arteries, which are pulsating strongly; tongue is coated with a dry yellow-brown fur; bowels are costive.

Fiat VS. statim ad ℥xiv.—Hydrarg. Submur., Pulv. Jac. veri, āā gr. v.; Mucil. Acaciæ q. s ut fiant pil. duæ statim sumendæ, et repetendæ horâ somni; post horas duas sumatur H. Sennæ.—H. Salin.; Liq. Antim. Tart. ʒss.; Syrupi ʒj. M. fiat haustus quartis horis sumendus.—Rep^r VS. vesperi ad ℥ xiv. si perstet dolor pectoris.—Diæta Febrilis.

25th, twelve o'clock.—The blood was cupped and slightly buffy: the venesection was not repeated. Bowels have been freely opened; tongue yellowish, brown, and dry; pulse 120, labouring. There has been some scanty expectoration of viscid, blood-streaked, but otherwise transparent, mucus. Inspiration is checked immediately by coughing, but not by pain. He can lie on both sides. Skin is hot and dry. Venesection was immediately repeated, and twenty-eight ounces of blood were taken away before the circulation became weakened. Shortly afterwards, the attempt to take a full inspiration was immediately checked by coughing.

Vesperi, si perstet tussis sicca rep^r VS.; augeatur dosis Liq. Antim. Tart. ad ʒij. in sing. haustu.—Rep^r pil. hac nocte.

26th.—The blood taken in the morning was very much cupped and buffy; that in the evening (amounting only to eight ounces)

was less so. Expectoration as yesterday; but he can now take a full inspiration, without any interruption by cough. Pulse 100, soft; tongue white and clammy; one evacuation; no nausea; skin still dry.

Rep^r Haustus Antimon.—Rep^r pil. horâ somni.

Nine o'clock P.M.—Skin is hot and dry; some cough, without pain, but no satisfactory expectoration.

Rep^r VS. statim ad ℥ x.

27th.—Blood very slightly cupped and buffy. Has had an easier night, but still the expectoration is very scanty and difficult; it is blood-stained, but otherwise transparent. Tongue is moist, skin also is moist; no nausea; no evacuation for the last twenty-four hours.

H. Sennæ statim; postea rep^r Haustus Antimon. quartis horis, et pil. horâ somni.

28th.—No nausea; bowels have been freely opened; skin is moist, but very warm; pulse ninety-six, soft; tongue is again quite dry, and brown in its centre, with yellow clammy edges; inspires fully, without either cough or pain, but still the expectoration is scanty and similar to that of yesterday.

Hydrargyri Submur., Ext. Conii, āā gr. iij. M. fiat pil. sextis horis sumend. —Rep^r H. Antimon.

30th.—Some little muco-puriform expectoration; skin moist, and moderately warm; tongue furred and pasty; two evacuations.

Rep^r medicamenta.

31st.—Last night, for the first time, vomited up the antimonial draught. Skin is natural; tongue moist, but still pasty; gums very slightly affected; some little transparent mucous expectoration.

Rep^r pil. omni nocte et mane.—Dimin^r dosis Liq. Antim. Tart. ad ʒj. in sing. haustu.

August 1st.—Is improving fast.

Omitt^r pilulæ et Haust. Antimon.—Acid. Sulph. dil. m. xv.; Syr. Tolutani, Mucilag. Acaciæ, āā ʒ ij.; Aquæ Cinnam. ℥ j. M. fiat haustus quartis horis sumend.—Sulphatis Magnesiæ ʒ iij. cras mane, nisi interim dejecerit alvus.

He went out cured, August 21st.

Case II. *Fever, with Cerebral Congestion.*—Ann Ryall, aged twenty-three, and single, was deposited at St. George's Hospital, in the evening of the 16th of August, almost in a comatose state, without any further information being left by her friends, than that she had been ill of fever for a week.

A pill of three grains of Calomel, with four grains of James's Powder, was immediately given; and a purgative draught, containing a scruple of Rhubarb, was given early in the morning.

August 17th, twelve o'clock.—She remains almost comatose, and incapable of answering questions. The present symptoms are—dilated pupils, scarcely contracting upon the approach of a candle; face and arms are flushed with a continuous scarlet efflorescence (not scarlatina) on the latter, but there is no appearance of it on the breast or lower extremities; there is slight superficial excoriation of the soft palate; the tongue is tremulous, dry, and brown; pulse is 120, very soft; abdomen is tumid, pressure upon it seems to excite uneasiness. Has passed a very scanty evacuation, resembling chopped spinach.

Adhibeatur Enema Catharticum statim et rep^r vesperi, nisi interim plenè soluta fuerit alvus.—Hydrargyri Submuriat., Pulv. Jac. veri, āā gr. iij.; Sacch. albi gr. vj. M. fiat pulvis quartâ quâque horâ sumendus.—Lotio Spirituosa capiti raso.—Diæta febrilis.

Half-past eight P.M.—Is now able to speak, and says that she has still some headache, but much less than before the cupping. Pulse 108, large, but very soft.

Applic^r Cuc. cr. nuchæ, et detrah^r sanguinis ℥viij. statim.—Rep^r pulvis.

18th, twelve o'clock.—The headache is much relieved, but face is still flushed, and the pupils are sluggish in contracting; pulse 108, of less size, but soft; abdomen is still tumid, but is pliant and no longer tender; there have been many copious, liquid, green stools; skin is hot and dry; she complains of great thirst, and the tongue is quite dry and brown.

Rep^r pulvis.

Nine o'clock P.M.—Pulse 108, soft; face is still flushed.

Rep^r Cuc. cr. &c. ad ℥ viij. statim.

19th.—Bowels have continued acting. Eyes now have their natural appearance, and the skin its natural temperature, but the tongue is still dry and foul. She is now quite rational, and states that she has still some pain just above the right ear. The gums are becoming slightly tumid.

Omit^r pulvis.—Haust. Salinus; Liq. Antim. Tart. ʒ ss.; Syrupi ʒ j. M. fiat haust. quartis horis sumendus.

20th, twelve o'clock.—Has had several liquid, dark-green evacuations during the night. Skin is warm and moist, but tongue is dry and brown; pulse 103, soft; the gums are slightly sore. She still complains of pain in the head.

Olei Ricini ʒ iij. statim sumendæ.—Rep^r Haust. Antimon.

Eight o'clock P.M.—The pain in the head continues.

Detrah^r nuchâ sanguinis ℥ x. statim ope Cuc. cr.

21st.—Head is less painful.

Rep^r Haust. Antimon.

23d.—The forehead feels hot, and the face is flushed; she complains of "great heat in the head;" the pulse is 108, and soft; tongue is cracked and furred in its centre, with red edges; the eruption is no longer observable; the evacuations have continued very dark-coloured and offensive.

Hydrargyri Submur. gr. v.; Pulv. rad. Jalapæ, Scammoneæ in pulvere, āā gr. vj.; Mucil. Acaciæ q. s. ut M. fiant pilulæ tres statim sumendæ.—Rep^r Haust. Antimon.

24th.—Five dark-brown evacuations. Skin moderately warm, but there is still some pain in the head; pulse 108, very small and soft.

Olei Ricini ℥ iij. statim.—Rep^r Haust. Antimon.

25th.—Six similar evacuations. Head is easier.

Olei Ricini ℥ iij. statim.—Rep^r Haust. Antimon.

26th.—The bowels acted frequently during the night, but the evacuations are now well coloured. Skin is of natural temperature; the scarlet efflorescence is again visible about the neck and face; tongue is quite moist and clean, but deeply fissured; pulse is very frequent.

Sulphatis Quinæ gr. ij. in formâ pilulæ quater die sumendæ. Superbibat haustum sequentum.—Acid. Sulph. dil. m. viij.; Syrupi Aurantii ʒj.; Mist. Camphoræ ʒxj. M.—Omittantur alia medicamenta.

27th.—Appetite has returned. One liquid but well-coloured evacuation.

Rep^r Pilulæ et Haustus.

September 2d.—P. cum Sulphatis Quinæ gr. iij. in sing. pilulâ.—Vin. alb. ℥ ij. quotidie ex aqua.

Under the continuance of this treatment she gradually recovered, and left the hospital September 26th.

In the first case, about three days after the invasion of the fever, the pulmonary affection, which previously was only a slight catarrh, was kindled up, by the febrile irritation, into an inflammation requiring the immediate adoption of depletory measures.

The progress of the case shows that copious and repeated venesection, though aided by powerful purgatives, and subsequently by considerable doses of tartarised antimony, failed to relieve the lungs from their inflammatory state, until the capillary vessels were brought under the control of mercury.

The most beautiful and convincing illustration of this power of mercury, when aided by venesection, to control the action of the capillary arteries, and thus subdue acute inflammation, is exhibited in inflammation of the iris, the transparency of the cornea affording the opportunity of witnessing not only the previous extent of the disorganising effects of the inflammation, but also each step in the progress of the counteracting influence of the remedy.

As soon as the mercury begins to affect the system, the progress of the inflammation is arrested,—the increased vascularity of the iris fades away,—the fine capillary vessels, resuming their natural size, cease to pour forth coagulable lymph; while that which had been previously effused is gradually absorbed, leaving the anterior chamber of the eye perfectly clear, and the iris in the full exercise of its proper functions,—provided that this remedy has been employed before the inflammation has produced any irreparable disorganisation of the part.

The observation of these facts naturally led the way to an earlier and a bolder administration of this remedy, in the treatment of acute inflammations of the internal viscera; for it seemed reasonable (due allowance having been made for the modifications produced in the progress and effects of inflammation by the variety of the structure inflamed,) to infer that the capillary vessels would be equally obedient in internal inflammations, as they had been seen to be in iritis, to the controlling influence of mercury.

Some interesting illustrations of these views may be found in Dr. FARRE's Preface to the second edition of SAUNDERS on Diseases of the Eye, (p. 38, 39;) and also in a short but very able paper on Iritis, by Mr. TRAVERS.

Further confirmations of the efficacy of mercury in subduing inflammation, are afforded by the various authors who recommend its exhibition in croup, hydrocephalus acutus, hepatitis, laryngitis, &c. &c.

In the same case it may be remarked that, during the exhibition of the tartarised antimony, half-grain doses did not produce vomiting, until there was some diminution of the inflammatory affection of the lungs. This is quite in accordance with the doctrine of the Controstimolo of the Italian practitioners, who maintain (and I believe most justly) that the impression made by a given quantity of any of this class of medicines on the system, varies inversely as the intensity of the existing inflammation: in order, therefore, to derive any considerable benefit from their exhibition, it is obviously necessary that the dose should be increased in proportion to the supposed activity of the inflammation. The capability of the patient to endure such a dose without experiencing nausea or vomiting, may, in some degree, serve as a test of the intensity of the inflammation.

In the second case, the appearance of the evacuations, resembling chopped spinach, and the comatose condition of the patient, indicating congestion of the brain, clearly demanded the free administration of purgatives; but it is probable that, if they had been continued beyond the 24th of August, the irritation produced in the intestines by their protracted use would have caused a continuance of unhealthy evacuations; which, however, under the use of mild doses of castor-oil, soon presented a healthy aspect.

*October 24th*, 1826.

